# Supplementary material for: Cloud BioLinux: pre-configured and on-demand bioinformatics computing for the genomics community
Source: BMC Bioinformatics. 2012 Mar 19;13:42. doi: 10.1186/1471-2105-13-42 (PMC3372431; doi:10.1186/1471-2105-13-42)
Supplement: Additional file 1 — Supplementary 1 Cloud BioLinux software documentation in the form of a mini, self-contained website. Users need to download and uncompress the .zip file, and open through a web browser the "index.html" file available on the main directory. (ZIP 1823 kb). [file 1471-2105-13-42-S1.ZIP › Cloud-BioLinux-Package-Documentation/docs/dnapenny.html]

Bio-Linux Software Documentation Pages

Back to search form

## dnapenny

|  |  |
| --- | --- |
| Name | dnapenny |
| Description | **dnapenny** is part of the PHYLIP package.  Copyright 1986-2004 by The University of Washington. Written by Joseph Felsenstein. Permission is granted to copy this document provided that no fee is charged for it and that this copyright notice is not removed.  **DNAPENNY** is a program that will find all of the most parsimonious trees implied by your data when the nucleic acid sequence parsimony criterion is employed. It does so not by examining all possible trees, but by using the more sophisticated branch and bound algorithm, a standard computer science search strategy first applied to phylogenetic inference by Hendy and Penny (1982). (J. S. Farris [personal communication, 1975] had also suggested that this strategy, which is well-known in computer science, might be applied to phylogenies, but he did not publish this suggestion).  **References:**  Felsenstein, J. 1993. PHYLIP (Phylogeny Inference Package) version 3.5c. Distributed by the author. Department of Genetics, University of Washington, Seattle.    Felsenstein, J. 1989. PHYLIP -- Phylogeny Inference Package (Version 3.2). Cladistics 5: 164-166. |
| Homepage | http://evolution.genetics.washington.edu/phylip.html |
| Remote Documentation | http://evolution.genetics.washington.edu/phylip/doc/dnapenny.html |
